# Accelerated Kidney Aging in Diabetes Mellitus

**DOI:** 10.1155/2020/1234059

**Published:** 2020-07-27

**Authors:** Jing Guo, Hui Juan Zheng, Wenting Zhang, Wenjiao Lou, Chenhui Xia, Xue Ting Han, Wei Jun Huang, Fan Zhang, Yaoxian Wang, Wei Jing Liu

**Affiliations:** ^1^Renal Research Institution; Key Laboratory of Chinese Internal Medicine of Ministry of Education and Beijing, Dongzhimen Hospital Affiliated to Beijing University of Chinese Medicine, Beijing 100700, China; ^2^Institute of Nephrology, and Zhanjiang Key Laboratory of Prevention and Management of Chronic Kidney Disease, Guangdong Medical University, No. 57th South Renmin Road, Zhanjiang, Guangdong 524001, China

## Abstract

With aging, the kidney undergoes inexorable and progressive changes in structural and functional performance. These aging-related alterations are more obvious and serious in diabetes mellitus (DM). Renal accelerated aging under DM conditions is associated with multiple stresses such as accumulation of advanced glycation end products (AGEs), hypertension, oxidative stress, and inflammation. The main hallmarks of cellular senescence in diabetic kidneys include cyclin-dependent kinase inhibitors, telomere shortening, and diabetic nephropathy-associated secretory phenotype. Lysosome-dependent autophagy and antiaging proteins Klotho and Sirt1 play a fundamental role in the accelerated aging of kidneys in DM, among which the autophagy-lysosome system is the convergent mechanism of the multiple antiaging pathways involved in renal aging under DM conditions. Metformin and the inhibitor of sodium–glucose cotransporter 2 are recommended due to their antiaging effects independent of antihyperglycemia, besides angiotensin-converting enzyme inhibitors/angiotensin receptor blockers. Additionally, diet intervention including low protein and low AGEs with antioxidants are suggested for patients with diabetic nephropathy (DN). However, their long-term benefits still need further study. Exploring the interactive relationships among antiaging protein Klotho, Sirt1, and autophagy-lysosome system may provide insight into better satisfying the urgent medical needs of elderly patients with aging-related DN.

## 1. Introduction

The increasing global morbidity of type 2 diabetes mellitus (T2DM) and chronic kidney disease (CKD) has provoked research efforts to overcome the growing prevalence of diabetic nephropathy (DN), which has been a global catastrophe due to limited efficacy with existing therapies and serious financial burden [1–3]. It is urgent to explore the unknown mechanisms underlying DN and discover curative efficient therapies.

As is well known, both T2DM and CKD are aging-related diseases. The morbidity of DM in people over 65 years old is more than twice that of people over 20 years old [1], and aging is a key factor attributing to nephron loss and resulting in CKD [4].

DM has been considered to be an inducer of accelerated cellular senescence and has been associated with aging-related cardiovascular diseases and kidney diseases due to high glucose levels [5]. However, the aging in a tissue-specific manner remains rarely explored. Globally, DM has been the leading cause of end-stage renal disease (ESRD), especially in elders [3, 6, 7]. Feasible histopathological patterns of individuals often imply the presence of other pathogenic factors, such as aging-related nephropathy, resulting in the complicated and difficult diagnosis and treatment of type 2 DN [8]. Thus, under the double risk factors of high glucose and aging, it is hypothesized that renal aging plays a vital role in the development of DN. Herein we will discuss current knowledge on renal aging-related mechanisms and potential therapeutic targets of DN.

## 2. The Role of Accelerated Kidney Aging in DN

Kidney aging is a complex process that interacts with many diseases, especially those that are more prevalent in the elderly population. Kidney aging is manifested in the decline of glomerular filtration rate (GFR), which is also the physiological characteristic of CKD [9, 10]. The GFR decreases by about 5%–10% per decade after 35 years of age, and the elderly, 70–75 years old, had 48% fewer intact nephrons than the younger patients aged 18–29 years old [11, 12]. It is often difficult to distinguish between chronological change and pathological changes, but some studies have focused on accelerated aging as a potential target to retard the process of renal diseases, including DN [13, 14].

In kidneys with premature aging due to the morbid state such as IgA nephropathy [13], the above characteristics may not be necessarily related to chronological change. For DN, the incidence of kidney disease in diabetic individuals partly depends on the aging-related nephron loss [15]. Except for the functional change of decreasing GFR, the structural changes are also observed as pathologic reduction in kidney size and renal histomorphology changes, including glomerulosclerosis, interstitial fibrosis, and tubular atrophy macroscopically and compensatory hypertrophy of renal cells, glomerular basement membrane (GBM) thickening, podocyte loss, and tubular epithelial cell (TEC) shrinking microscopically [16].

**Figure 1:**
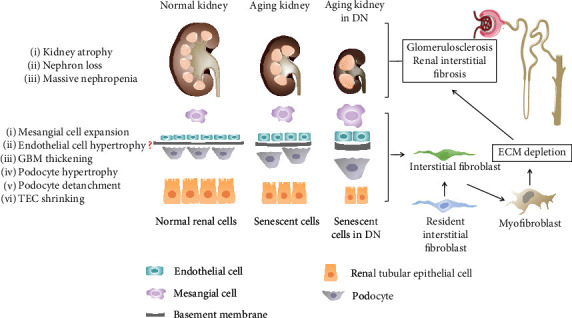
Normal kidney, kidney aging in nature, and kidney aging under DM conditions. Each normal kidney possesses thousands of nephrons. With aging and the onset of DN and the interaction of the two, nephrons are gradually lost and become massive, particularly when occurring in aging kidneys with underlying DM. Macroscopically, pathologic reduction is observed in kidney size and renal histomorphology changes, including glomerulosclerosis, interstitial fibrosis, and tubular atrophy. Microscopically, compensatory hypertrophy of renal cells, glomerular basement membrane (GBM) thickening, podocyte loss, and tubular epithelial cell (TEC) shrinking are seen, which contribute to driving an associated dysfunction like the pathologic changes in kidneys as mentioned above.

It has been demonstrated that the kidney appears aging phenotype which represents a proximate mechanism by which the kidney is damaged in DN [17, 18], leading to a complicated and difficult diagnosis and treatment of type 2 DN. Thus, accelerated kidney aging may be an important part of the pathogenesis of DN (Figure 1). However, the cellular and molecular mechanisms of kidney aging in diabetic individuals are complicated and poorly understood.

## 3. Factors Associated with Accelerated Kidney Aging in DN

DN is the renal manifestation of the hyperglycemia-driven process in vulnerable sites along with retinopathy. Multifactors are posed with DN beyond high glucose, such as oxidative stress and activation of the renin-angiotensin-aldosterone system (RAAS) [15]. Accumulation of advanced glycation end products (AGEs) [19] and inflammation also contribute to the process of DN [20]. Similarly, kidney aging is associated with clinical factors such as DM and hypertension, as well as tissue factors including angiotensin II, AGEs, oxidative stress, and so on [21]. Hence, herein we focus on AGEs, hypertension, inflammation, and oxidative stress in diabetic kidneys.

AGEs, which are accumulated in multiple tissues during aging, provide information of a nonenzymatic reaction of proteins and carbohydrates that can be detected with an increased level in each related organ of patients with DM [22]. AGEs, as a result of pathologically increased glycation due to chronic hyperglycemia, have a variable “pathological expression” in DM, kidney failure, and tissue aging [23]. Interestingly, reducing AGEs extends the lifespan of yeast [24]. Hence, AGEs can be a biomarker of aging and may lead to renal lesions in DM related to kidney aging [25]. In hyperglycemic circumstances, the accumulated AGEs induce the accelerated aging of kidney dysfunction by inducing podocyte damage and apoptosis of mesangial cells and the expression of transforming growth factor-*β* (TGF-*β*), the latter of which plays a pivotal role in fibrogenesis [26, 27]. AGEs stimulate the activation of the receptor for AGEs (RAGE), which induces oxidative stress and cellular dysfunction. In the kidney, the RAGE-AGE activation also contributes to the induction of oxidative stress, endoplasmic reticulum (ER) stress inflammatory, and fibrotic responses by activating different intracellular signaling pathways, such as phosphatidylinositol 3 kinase/protein kinase B (PI3K/Akt), mitogen-activated protein kinase/extracellular regulated protein kinases (MAPK/ERK), and nuclear factor kappa-B (NF-*κ*B), all of which lead to functional and structural damages of kidneys, as well as premature aging [19, 28].

Hypertension is one of the clinical symptoms of DN due to the dysfunction of the RAAS, and RAAS inhibitors have been considered to be the most effective therapy for DN [29]. In the aging kidney, the RAAS is related to glomerular and tubular damage via oxidative stress and/or downregulating antiaging proteins, such as Sirtuins and Klotho [30, 31]. AGEs stimulate angiotensinogen production in renal proximal tubular cells, which results in inappropriate activation of RAAS and exacerbates the development of diabetic-related kidney lesions [32]. Thus, these findings suggest that the abnormal activation of RAAS aggravates kidney damage and may lead to accelerated senescence in DN.

Oxidative stress (OS) is considered a major factor in the pathogenesis of DN due to its contribution to hyperglycemia and hypertension [33]. OS contributes to aging as a result of causing increased damage to important cellular targets, increasing mutation rates, and inducing growth inhibition [34]. In diabetic kidneys, the main sources of OS include nicotinamide adenine dinucleotide phosphate (NADPH) oxidase activation [35], mitochondrial dysfunction [36, 37], xanthine oxidase pathway abnormality, cyclooxygenase pathway dysregulation, and endothelial nitric oxide synthase uncoupling [38]. These sources of OS contribute to a range of harmful intracellular events, including DNA damage within the nucleus and mitochondria, and ultimately result in the death of renal intrinsic cells via apoptosis that ER stress and cellular senescence contribute to [39, 40]. Additionally, high glucose- (HG-) induced reactive oxygen species (ROS) production results in increased TGF-*β*1 expression, which is the core link of renal fibrosis in diabetic kidneys and results in the epithelial-mesenchymal transition [41]. Recent studies have reported that P66 plays a key role in the pathogenesis of DN because of its relation to OS. ROS metabolism was substantially increased in HG-induced mesangial cells in association with more cell death via apoptosis, as well as the acquisition of a senescent phenotype and a homozygous mutation at the P66 locus that confers delayed aging phenotypes in the diabetic kidneys [18]. The abnormal expression of p66 may have a correlation with the protein kinase C (PKC) *β* activation and then regulate NADPH oxidase, which could further promote the activation of OS [42].

Inflammation is the both cause and consequence of accelerated aging leading to renal damage [43, 44]. Inflammation plays an important role in the genesis of DM as well as in the development of diabetic complications, including DN. The metabolic alterations with the accumulation of toxic products, such as AGEs and hemodynamic factors and the activation of RAAS, enhance inflammation of the kidney [45, 46]. Excessive ROS production in kidney tissues activates inflammation-related signaling pathways, such as PKC, MAPK, and NF-*κ*B, and leads to the production of a large number of cytokines and growth factors that trigger the onset of DN. This process then causes the deposition of the extracellular matrix (ECM) in glomeruli, the differentiation of tubular epithelial cells (TECs), and the interstitium and an increase in the synthesis of glomerular fibronectin (FN) proteins in the mesangial region [47]. Proteinuria, a marker of renal lesions, aggravates the local microinflammatory response and enhances interstitial cellular infiltration, leading to the overexpression of mesangial matrix production, glomerular basement membrane (GBM) thickening, and glomerulosclerosis [48]. Persistent microinflammation is increased in the aging kidney and provides potential mechanistic links between the epigenetic landscape of aging and renal dysfunction [49, 50].

**Figure 2:**
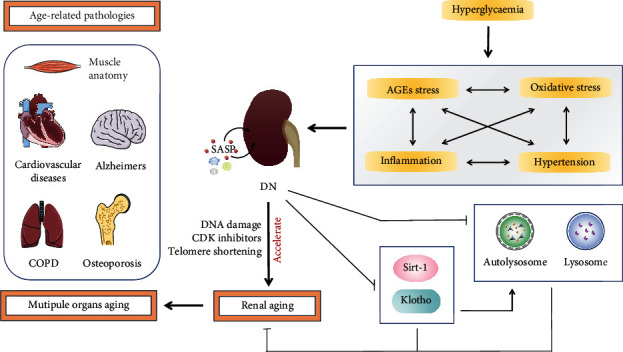
Schematic of accelerated kidney aging in DM and the relationship between kidney aging and systemic aging. In diabetic kidneys, AGE accumulation, oxidative stress, inflammation, and hypertension caused by hyperglycemia-induced metabolic impairment are central to the development and progression of DN and, hence, the acceleration of defining renal aging. In addition, the senescence of DN plays a key role in aging-related pathologies, such as cardiovascular diseases, Alzheimer's, chronic obstructive pulmonary emphysema (COPD), muscle atrophy, and osteoporosis.

Taken together, in diabetic kidneys, multiple stresses, such as AGEs accumulation, hypertension, oxidative stress, and inflammation, induce a negative environment that accelerates senescence that is manifested in renal functional decline and aberrant structural changes (Figure 2).

## 4. Cellular Senescence and Kidney Aging in DN

It has been reported that cellular senescence plays a vital role in the aging and diseased kidney [51]. Cellular senescence can be widely observed in the aging kidney as an important cellular process that contributes to age-related kidney changes and CKD progression [52]. Besides, it is also involved in kidney aging. During aging, cellular senescence can be independently induced by stresses such as oxidative stress, which is called stress-induced premature senescence through p16^INK4^/retinoblastoma (Rb) or p53/p21^Cip1^ pathway. In kidneys, senescent cells are manifested in the arrest cell cycle, imbalance of apoptosis and proliferation, and senescence-associated secretory phenotype (SASP), leading to kidney aging by increasing sensitivity to injury and reducing repair after injury [53, 54]. In addition, clearance or depletion of senescent cells can relieve age-related damage and dysfunction in kidneys [51]. The conversion of a senescent phenotype is a significant step that underlies the pathogenesis of renal lesions as an early response to DM, which might be a target to retard DN progression [55].

Senescent cells provoke permanent cell cycle arrest by triggering cyclin-dependent kinase (CDK) inhibitors which mediate renal injury in DN. Studies on both progeroid and naturally aged mice showed that selective elimination of p16^INK4^-expressing senescent cells increased health and lifespan [56, 57], and in humans, p16^INK4^was shown as one of the top genes exhibiting elevated expression with age in multiple tissues, including kidneys [58]. In diabetic kidneys, accelerated senescent phenotypes were mainly observed in tubular cells and podocytes, and the positive correlation between glomerular p16^INK4^ with proteinuria indicated that glomerular cellular senescence takes partly responsibility for altered permeability [17]. AGE-induced p16^INK4^ expression and premature senescence were successfully relieved by an ER stress inhibitor and cyclic AMP-dependent transcription factor (ATF) 4 gene silencing in TECs of DN [14]. The expression of p21^Cip1^ was increased with an upregulation in senescence-associated *β*-galactosidase (SA-*β*-gal) staining in TECs [55], and the changes could be suppressed by insulin therapy. However, senescence induced by high glucose was inhibited in p21^Cip1^ knockdown mice, indicating that aging in renal TECs is mediated by a p21^Cip1^-dependent pathway [59]. TGF-𝛽1 induces p21^Cip1^ dependent hypertrophy of mesangial cells and plays an important role in the pathogenesis of chronic kidney diseases, including DN. TGF-𝛽1 increases p21^Cip1^ gene expression in renal mesangial cells and elevates the recruitment of the H3K4 methyltransferase SET7/9 to the p21^Cip1^ gene promoter [60]. It has been reported that there exists a complex crosslink between ER stress and p21^Cip1^ signaling in aging-related diseases [61]. In diabetic TECs, the ER stress marker is expressed at a higher level compared to that of controls and is positively correlated with enhanced SA-*β*-gal-positive cells and colocalization with RAGE. ER stress-mediated premature senescence is dependent on p21^Cip1^ activation, because the ER marker and p21^Cip1^ were colocalized in the same diabetic TECs in vivo and in vitro. Moreover, inducers of ER stress directly cause premature senescence of TECs by p21^Cip1^ activation. Thus, p21^Cip1^ signaling plays a deterministic role, which is promoted by RAGE, in the premature senescence of TECs that is mediated by the activation of ER stress [62]. P27^Kip1^ is another member of CDK2 inhibitors. Chronic hyperglycemia induces hypertrophy and damage to podocytes and mesangial cells related to p27^Kip1^ [63, 64]. In Type I DM, p27^Kip1^ knockout mice exhibited milder renal lesions compared to that of p27^Kip1 +/+^mice due to the regulation of TGF-*β*; additionally, angiotensin receptor blocker treatment alleviated renal hypertrophy by inhibiting p27^Kip1^ expression [64, 65]. Senescent phenotypes might have individualized expression in different renal cells, but controversial results have still been reported. For example, some studies showed that p16^INK4^ expression increased in diabetic TECs, whereas other studies have reported that overexpression of p21^Cip1^ but not other CDK inhibitors, such as p16^INK4^ and p27^Kip1^, is increased due to hyperglycemia [59]. These discrepancies might be related to the different stages of DN in different studies. However, further studies still need to further account for these discrepancies in the future.

Another feature of senescent cells is senescence-associated secretory phenotype (SASP). SASP is a significantly distinctive feature of senescent cells that includes diverse cytokines, chemokines, growth factors, proteases, and lipids, which may promote inflammation in aging-related diseases [66]. NF-*κ*B is activated by DDR and p38 AMP-activated protein kinase (AMPK) in the production and secretion of SASP, and the main components of SASP during the mature period are soluble cytokines, such as C-X-C-motif chemokine ligand-1/2 (CXCL-1/2), interleukin- (IL-) 8, IL-1, matrix metalloproteinases (MMPs), and ECM proteins, that may contribute to the accumulation of ECM and renal interstitial fibrosis in CKD [66]. Premature senescence of intrarenal and extrarenal cells appearing with the overexpression of SASP leads to aggregative kidney aging and disease progression. The similarities of SASP and the CKD-associated secretory phenotype (CASP) have been compared with one another and may present a link between CKD and renal cellular senescence [67]. Considering the microinflammation in the mechanisms of DN, we summarize the relative specific secretory phenotypes in DN compared with those in CASP (Table 1).

Telomere shortening is another cause of triggering senescence. It has been shown that telomeric DNA is lost in the aging kidney of humans [68], and the shortening of telomere length may be associated with CKD occurrence and/or decline of kidney function [69]. In cultured TECs, high glucose accelerated telomere shortening may be mediated by oxidative stress because of hyperglycemia [17]. For mesangial cells, senescence was associated with telomere attrition induced by high glucose via the p53-p21-Rb signaling pathway. [70]. These results may contribute to a new strategy for the treatment of DN.

## 5. Main Cellular and Molecular Mechanisms of Accelerated Kidney Aging in DN

Molecules and signaling pathways related to the mechanisms of accelerated kidney aging in DM remain multiple and complicated. Herein, we review the mechanisms in four aspects (including Sirt1, Klotho, autophagy, and lysosome) because of their core and inevitable role related to the kidney aging in DM.

### 5.1. Sirt1 and Kidney Aging

**Figure 3:**
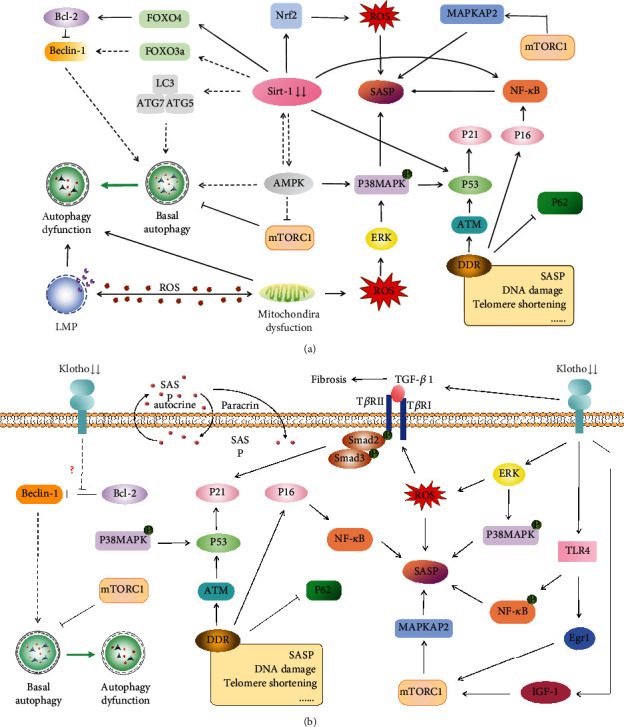
Klotho, Sirt1, and lysosome-dependent autophagy in DN. Under physiological conditions, the antiaging protein klotho and Sirt1 can enhance basal autophagy, protecting the kidney from DN and aging. Under DM conditions, the expression of Klotho and Sirt1 in the kidney is downregulated. Besides, the autophagy is not able to maintain cellular homeostasis and resist renal cell senescence. This overwhelmed suppression in DN accelerates kidney aging. The *solid lines* with *black arrowheads* indicate promoting function. The *solid lines* with *black truncated ends* stand for inhibiting function. The *dotted lines* in *black* show the disturbed and altered regulatory function in the diabetic kidney with aging. The *solid line* in *green* represents autophagy from normal to dysfunction. Abbreviations: DDR: DNA damage response; SASP: senescence-associated secretory phenotype; ROS: reactive oxygen species; ATM: ataxia telangiectasia mutated; MAPK: mitogen-activated protein kinase; ERK: extracellular signal-regulated kinase; NF-*κ*B: nuclear factor-kappa B; Nrf2: nuclear factor E2-related factor 2; SIRT1: sirtuin 1; AMPK: adenosine monophosphate–activated protein kinase; mTOR: mammalian target of rapamycin; LC3: microtubule-associated protein 1A/1B–light chain 3; Atg5: autophagy-related 5; Atg7: autophagy-related 7; BCL2: B-cell lymphoma 2; P62: SQSTM1/sequestosome 1; FOXO3a: forkhead box O3a; FOXO4: forkhead box O4; TGF-*β*: transforming growth factor-*β*; LMP: lysosomal membrane permeabilization.

Sirt1 protein expression can be detected in the normal kidney, and its expression level is decreased in the diabetic kidney [71, 72]. The decline of Sirt1 expression in diabetic-kidney tissue leads to mitochondrial damage and OS and plays a vital role in renal premature senescence by impairing antistress capacity and accumulating renal lesions [73]. The podocyte-specific loss of Sirt1 not only aggravates diabetic kidney injury but also leads to aggravated aging-induced glomerulosclerosis and albuminuria. This phenomenon is associated with reduced activation of transcription factors, such as peroxisome proliferator-activated receptor- (PPAR-) *α* coactivador-1 (PGC-1*α*)/PPAR*γ*, Foxo3, Foxo4, and p65 NF-*κ*B, via Sirt1-mediated deacetylation [71](Figure 3(b)).

Caloric restriction was first proposed to be associated with antiaging in 1934, and Sirt1 has been reported as an antiaging molecule related to caloric restriction of aging-related diseases [74, 75]. In fatty diabetic Wistar rats, dietary restriction ameliorated renal abnormalities and decreased expression of Sirt1, increased expression of acetylated-NF-*κ*B, and impaired autophagy. These results demonstrated that dietary restriction-mediated Sirt1 restoration exerted anti-inflammatory effects and improved autophagy dysregulation, which resulted in the amelioration of renal injuries in DM [76]. AGEs reduced the Sirt1 level but enhanced the expressions of FN and TGF-*β*1 in mesangial cells. The overexpression of Sirt1 further increased the nuclear accumulation of nuclear factor E2-related factor 2 (Nrf2) and promoted heme oxygenase 1(HO-1) and superoxide dismutase (SOD) 1 levels, whereas it decreased ROS, FN, and TGF-*β*1 levels induced by AGEs (Figure 3(b)). Thus, Sirt1 showed resistance against oxidative stress-mediated diabetic renal fibrosis [77]. The same results could also be demonstrated in human renal TEC line HK2 cells [78]. Sirt1 regulates mitochondrial biogenesis and turnover in relation to the deacetylation of PGC-1*α* [79]. HG accelerated mitochondrial dysfunction and downregulated Sirt1 expression. Activation of the Nrf2- antioxidant response element (ARE) antioxidative pathway ameliorates hyperglycemia-mediated mitochondrial dysfunction partly through Sirt1 [80–82] (Figure 3(b)).

These findings highlight that Sirt1 plays an important role in antisenescence in DN, by targeting members of SASP (e.g., NF-*κ*B and TGF-*β*), as well as by relieving renal inflammation, fibrosis, and oxidative stress to alleviate mitochondrial damage of renal cells.

### 5.2. Klotho and Kidney Aging

Klotho is an aging suppressor gene that encodes a single-pass transmembrane protein with an extracellular portion, exhibits multiple pleiotropic effects, and is found in two forms: an intermembrane form and a secreted form [83]. The membrane klotho forms a complex with fibroblast growth factor 23 (FGF23) receptor, and this complex mediates phosphate homeostasis and vitamin D metabolism. Secreted Klotho acts as a humoral factor targeting distant organs with pleiotropic activities consisting oxidative stress regulation, growth factor signaling, and ion homeostasis and can be detected in blood, urine, and cerebrospinal fluid [84, 85].

In mice, the overexpression of Klotho extends lifespan, whereas mutations to the Klotho gene shorten lifespan. In humans, serum Klotho levels are lower in individuals that are 40 years old or older [86]. Klotho is predominantly expressed in renal TECs and is equipped with a variety of biological functions. The antiaging role of Klotho is related to its downregulation of cytokines and growth factor signaling, such as interferon-*γ* (IFN-*γ*), insulin-like growth factor-1 (IGF-1), and TGF-*β*, and works by inducing antioxidative stress mediated by insulin, via IGF-1 signaling [86] (Figure 3(a)). Furthermore, it is related to iron imbalance [85]. The level of Klotho expression in diabetic patients and mice was significantly reduced, which was related to increased urinary calcium excretion [87]. Klotho levels were decreased with increasing albumin excretion in patients [88] and were significantly associated with a decline in eGFR [89]. These findings are similar to those of other studies [90]. Thus, Klotho may be a predictive biomarker for the progression of DN.

Klotho was observed to attenuate renal fibrosis in DN. The expression of Klotho in renal tubules declined in streptozotocin- (STZ-) diabetic rats, and Klotho alleviated HG-induced profibrotic genes, TGF-*β* signaling, and cell hypertrophy in rat renal fibroblast cell line NRK-49F cells. Moreover, Klotho attenuated HG-induced FN expression and cell hypertrophy via ERK1/2 and p38 kinase-dependent pathways [91] (Figure 3(a)). In addition, in spontaneously diabetic mice, it was also observed that the upregulation of Klotho attenuated renal hypertrophy, albuminuria, glomerular mesangial expansion, as well as attenuated glomerular macrophage infiltration and suppressed proinflammatory cytokines [92]. Additionally, Klotho downregulated early growth response factor 1 by inhibiting TGF-*β*1/Smad3 signaling in HG-induced human mesangial cells to combat renal fibrosis [93] (Figure 3(a)).

Klotho has also been shown to be involved in anti-inflammation in DN. In db/db mice, renal Klotho gene and protein expression were significantly downregulated, and overexpression of Klotho repressed NF-*κ*B activation and subsequent production of inflammatory cytokines in response to TNF-*α* stimulation. These findings suggest that Klotho serves as an anti-inflammatory modulator that negatively regulates the production of NF-*κ*B linked inflammatory proteins [94] (Figure 3(a)).

Klotho deficiency may be associated with increased OS, and anti-OS is a potential treatment target for DN. OS is more serious in patients with DN compared to that of the healthy controls [95]. AGE-triggered cellular senescence was at least partially due to the activation of OS, and Klotho overexpression protected TECs from injury induced by AGEs and H_2_O_2_; thus, Klotho can attenuate the OS [96].

Klotho inhibits the progression of DN by attenuating vascular endothelial dysfunction, calpain activation, and chronic inflammation. In addition, Klotho has been shown to inhibit diabetic renal tubular hypertrophy by inhibiting IGF-1 signaling [92, 97] (Figure 3(a)). Taken together, Klotho participates in multiple antiaging pathways to protect renal function in DN.

### 5.3. Autophagy and Kidney Aging

Autophagy is a degradation and recycling system in the process of growth, development, and aging. Autophagy has two main roles in the cell: (1) self-digestion in nutrient-deficient conditions to achieve the reuse of energy; (2) degradation of damaged or excess organelles and macromolecules to maintain cellular metabolism under stress [98]. A large number of studies have demonstrated that autophagy inhibition increases with aging and progresses in aging-related diseases [99, 100]. As a convergent mechanism of multiple longevity models, the activity of basal autophagy is elevated in many longevity paradigms of lifespan extension or delayed aging [101]. Autophagy appears to be a causal effector of existing antiaging manipulations such as the longevity drugs, such as resveratrol, rapamycin, and spermidine [102]. Rapidly accumulating evidence has revealed that autophagy is involved in renal physiology, kidney aging, and several kidney diseases, and plays a renoprotective role in various animal models.

Notably, serum levels of Beclin-1, a regulator of autophagy, are reduced in patients with DM and DN. Beclin-1 is also related to the stage of DN and correlates with the degree of albuminuria, which indicates autophagy inhibition in patients with diabetic renal lesions [103]. In addition, the basal activity of autophagy is inhibited in intrinsic cells in diabetic kidneys [104]. The potential mechanisms might be due to the appearance of oxidative stress and inflammation secondary to stimuli such as AGEs and urinary proteins [105–107]. Autophagy regulates TGF-*β* expression and suppresses kidney fibrosis through the autophagic degradation of mature TGF-*β* [108], which contributes to the occurrence of diabetic diffuse glomerulosclerosis and the excessive deposition of fibrotic materials in DN [109]. The LC3-II/LC3I ratio, Atg5 level, and Atg7 expression in the diabetic kidney with Sirt1 knockdown sharply declined [110], whereas Sirt1-induced autophagy was enhanced in an experimental model of DN [111]. Additionally, other studies have indicated that AMPK downregulation [112] and mTORC1 upregulation [113] are two key players in orchestrating events in autophagy and aging, which are crucial for the onset or progression of DN [114, 115].

Impaired autophagy has been demonstrated in vivo in DN patients that LC3 and P62 accumulation in the kidney of DN patients [116] and DN animal models [117], as well as in vitro [106]. Actually, the antisenescence of autophagy remains controversial. Oxidative stress-induced senescence is linked to autophagy impairment [118]. Mitochondrial dysfunction induced by high glucose is the main cause of oxidative stress and triggers senescence of TECs which can be modulated by mitophagy [119]. It was reported that atg5-deficient podocytes developed a series of aging-related alterations, such as lipofuscin accumulation and damaged mitochondria increase, the load of oxidized proteins, and the occurrence of ubiquitin and p62/SQSTM1-positive protein aggregates [120]. However, another study illustrated that silencing atg-5 reduced the hallmarks of stresses-induced TEC senescence [121]. It was clarified that general autophagy played an antisenescence role, but under stresses, once cells over a certain time point in senescence, autophagy showed prosenescence because of removing stresses that senescent cells must treat with [122]. More interestingly, it was reported that increased lysine63 ubiquitination not the whole level of ubiquitin was related to impaired autophagy and apoptosis of TEC induced by hyperglycemia [116]. So how autophagy in DN influences cell fates remains complicated. For atherosclerosis, defective autophagy promoted senescence and apoptosis in endothelial cells. Defects in autophagic machinery seemed to initiate apoptosis, while the expression of p53 was likely to onset senescence. Anyhow, senescence and apoptosis were two complementary cell fates controlled by autophagy [123]. Nevertheless, in DN, more studies are needed to conduct to elucidate how autophagy promotes cell fates.

### 5.4. Lysosome and Kidney Aging

Lysosomes are the main catabolic organelles essential for cell homeostasis that are found in all animal cell types except for erythrocytes and play a pivotal role in regulating a variety of processes, such as calcium signaling and nutrient responses to autophagic degradation of intracellular components. Lysosomes have been reported to have important significance in the control of lifespan [124]. Specifically, lysosomal dysfunction induces failure of cellular homeostasis during aging, which reduces the overall degradative capacity of cells and influences cellular and organismal life and death [124, 125]. Cellular senescence is partly determined by the lysosomal function related to mitochondria. Lysosomal dysfunction induces the imbalance of mitochondrial turnover, resulting in the generation of more ROS, which in turn targets lysosomes [126]. In ROS-senescence, mitochondrial dysfunction plays an initiating role, while lysosomal dysfunction is more directly responsible for senescence [118].

It is well established that lysosomal-cathepsins translocation, caused by lysosomal membrane permeabilization (LMP) (Figure 3), induces lysosomal-dependent cell death (LDCD), which has been observed in some age-related diseases, such as Parkinson's disease [127, 128]. In the study of DN, renal TECs with AGEs stimulation triggered the lysosomal membrane permeabilization, resulting in a decrease of activity of cathepsin B and cathepsin L, lysosomal acidification, and defective degradation of DQ-ovalbumin. However, these effects of AGEs can be blocked by antibodies against AGE-specific receptors or by antioxidants, which indicates that in the condition of DN, oxidative stress may play an important role in lysosomal dysfunction and further lead to tubular cell senescence and apoptosis [106]. Furthermore, urinary LAMP-2 levels are significantly decreased in patients with DN, which correlated with the urinary albumin to creatinine ratio (ACR) and GFR and might lead to the accumulation of autophagic vacuoles [129]. Lysosomal cathepsins are responsible for initiating and executing cell death during aging [130] (specifically in the kidney), and dysregulation of cathepsins B, D, L, and S is shown to be responsible for the onset or progress of kidney diseases [131]. Altered cathepsin D was captured in the tubulointerstitium of renal tissue from patients with DN, and, more precisely, cathepsin D upregulation suppressed the LMP and loss of mitochondrial membrane potential triggered by AGEs, which suggests a protective role in DN [132].

Recent data unveil mTOR activation, nuclear translocation of transcription factor EB (TFEB) inhibition, and the interaction between mTOR and TFEB in glomeruli from db/db mice and podocytes treated with AGEs, which is an irreplaceable factor that involved in the pathogenesis of DN [133]. Previous work has confirmed that TFEB is the master gene in coordinating lysosomal expression and its regulated network [134] and regulates lysosomal biogenesis and cellular clearance [135, 136]. Multiple lines of evidence indicate that mTORC1 resides at the lysosomal surface [135], in response to nutrient-sensing pathways [137], and accelerates cellular and organismal senescence [138, 139]. Hence, in the context of aging and longevity, it will be captivating to eavesdrop on the “cross-talk” between kidney disease and aging through lysosome-based signaling pathways.

## 6. Main Consequences of Accelerated Kidney Aging in DN

There are two main consequences from the accelerated kidney aging in DN. First, more rapid GFR declines. Kidney aging is featured with the reducing GFR. Due to chronic hyperglycemia, hypertension, and proteinuria, 34.8% of patients have a progressive disease with an annual GFR decline of 3.57 ± 1.45 mL/min/1.73 m^2^/year due to chronic hyperglycemia, hypertension, and proteinuria [140]. Significantly, DN patients with heavy proteinuria experience a rapid renal deterioration with the rate of decline of 46–60 mL/min/1.73 m^2^/year [141]. Those represent a more accelerated decline compared to normal biological aging at an average rate of 1 mL per year after 30 years old [142]. Second, kidney aging triggers system aging, contributing to the mortality of DN. Patients with DM and CKD have a sharply higher risk for cardiovascular diseases (CVD) compared to diabetic patients with no CKD and CVD, not ESRD seems to be the main cause of death [143]. In Japan, DN has been the main cause of chronic hemodialysis and in populations with chronic hemodialysis, the morbidity of frailty in the DN group was significantly higher than that in the non-DN group [144]. Alarmingly, 32.7% of individuals with DN have frailty significantly increasing the risk of developing ESRD and mortality [145]. A variety of other phenotypes of premature aging still have been observed in patients with CKD, such as the following: vascular calcification, cardiac insufficiency, osteoporosis caused by calcium and phosphorus metabolism disorders, muscle atrophy, and cognitive dysfunction [43, 146, 147]. As a specific CKD, aging-related DN is closely related to systemic aging. A range of factors play a part role in the aging-related decline in renal function, including increased levels of oxidative stress and inflammatory reactions, activation of the RASS, and the stress resistance responses, excessive secretion of angiotensin II, mTOR overactivation, deficiency of Klotho and vitamin D [21, 44]. These factors mentioned above could have a role in impairing the antiaging pathway and may underlie premature aging in DN [96, 148–150]. In 852 healthy adults aged 30–98 years, Han et al. reported that declines of naturally aging-related renal function and cardiac diastolic function are not independent processes [151]. In addition, sarcopenia in elderly patients with kidney diseases had a higher prevalence compared with that of younger ones [152], which may provide a better understanding that kidney aging accelerates systemic multiple organ dysfunction.

## 7. Potential Therapeutic Strategy Targeting Accelerated Kidney Aging in DM

Animal models have shown that diet interventions retard systemic and kidney aging, especially diets with low-AGE contents and enrichment of antioxidants [153]. A 40% adult-onset calorie restriction tended to suspend the age-related structural alterations of kidney-like glomerulosclerosis, interstitial-fibrosis formation, and vascular-wall thickening, which was associated with the decrease of the accumulation of mitochondrial enzyme abnormalities [154]. Short-term caloric restriction was demonstrated to play a protective role against renal senescence via increasing autophagic activity and reducing oxidative stress [155], which may be related to the modulation of AMPK/mTOR signaling [156], attenuating inflammatory process via downregulation of NF-*κ*B [157], as well as the suppression of apoptosis [158]. For humans, some clinical studies have reported the effects of diet restriction for diabetic kidneys. Calorie restriction exhibits renoprotection via amelioration of glomerular hyperfiltration of patients with T2DM with abdominal obesity [159]. With a period of four months of low protein-diet intervention, renal function with a restricted glucose control improved among diabetic patients with macroalbuminuria [160]. However, there is a lack of evidence to show whether there is a beneficial effect of long-term diet restriction for diabetic patients with kidney lesions [161].

Considered a caloric restriction mimetic, resveratrol, a natural polyphenol extracted from grapes and several plants, is characterized as a powerful free-radical scavenger and antioxidant and has been recognized to have an effect on antiaging and life extension. A clinical trial showed that resveratrol might be an effective complementary selection with ARBs to reduce albuminuria in patients with DN [162]. Animal experiments have illustrated that resveratrol restrains the oxidative stress markers in diabetic rats, decreased the expression of renal TGF-*β*1 and FN [163], which might be associated with the activation of Sirt1 and PGC-1*α* [164], and increased the expression of Foxo1 [165] and PPAR*δ* [166]. Multiple signaling pathways, including PI3K/Akt, c-Jun N-terminal kinase (JNK)/NF-*κ*B, Akt/NF-*κ*B, and p38 MAPK/TGF-*β*, have been demonstrated to underlie the renoprotective mechanisms of resveratrol [167] and its antiaging effect in diabetic kidneys [168]. Interestingly, it has been reported that resveratrol has the potential capacity to increase the expression of antiaging proteins such as Klotho and Sirt1 to alleviate the vascular calcification in CKD.

Metformin application remains controversial for DN due to its risk on hyperlactatemia and renal impairment in moderate-to-severe CKD, especially in the elderly [169, 170]. Actually, dosage adjustment of metformin appears to be safe and efficacious for moderate-to-severe CKD, and evidence has shown its potential benefits in lowering the risk of death and cardiovascular event in stage 3 CKD and sustaining calcium-phosphorus homeostasis to prevent vascular calcification [171, 172]. Metformin has shown antiaging benefits in diseases including DM and CKD [173–175] and in reducing the all-cause mortality and diseases of aging, including tumors and cardiovascular diseases of diabetics, even compared to nondiabetic [176]. The activation of AMPK plays a vital role in the mechanisms underlying the beneficial effects of metformin for DN. Hyperglycemia gave rise to suppression of phosphorylation and activity of AMPK, leading to multiple pathophysiological changes [177]. Metformin has elucidated the protective effect on podocytes, glomerular mesangial cells, and proximal tubular epithelial cells. In vitro, metformin showed antiapoptosis of podocytes induced by high glucose due to activation of AMPK and inhibition of mTOR signaling [178], and the activation of AMPK seemed to be associated with the activation of P2 receptors via upregulation extracellular ATP concentration [179]. Additionally, metformin adjusted nephrin protein expression [180] and repressed oxidative injury to restore podocytes [181] and also relieved insulin resistance of podocytes through activating Sirt1 and AMPK in diabetic rats [182]. Metformin alleviated inflammation of mesangial cells [183], which was related to upregulated glucagon-like peptide-1 (GLP-1) receptor expression [184]. The Sirt1/Foxo1 signal pathway was focused on demonstrating the antioxidative stress effect of metformin subsequently with the activation of autophagy in diabetic rats and high-glucose-induced mesangial cells [185, 186]. Increasing PGC-1*α* expression in high-glucose-induced TECs and suppression of AKT and mTOR activation in proteinuria induced TECs, subsequently followed by augmented autophagy and mitochondrial dynamics or ER stress that contributed to the renoprotective effects of metformin in DN [187, 188]. Meanwhile, metformin and rapamycin reversed high-glucose-induced premature senescence of renal cells, as well as induced downregulation of Connexin43 via activation of AMPK and the inhibition of mTOR [189], while the P21 expression was suppressed via modulation of AMPK by metformin-independent repression of mTOR [190].

It is important to note that not only metformin but other antidiabetic agents show emerging renoprotection targeting renal cellular senescence. It has been reported that in patients with type 2 diabetes and kidney disease, compared to a placebo, the inhibitor of (sodium-glucose cotransporter-2) SGLT2, canagliflozin, indeed decreased the relative risk of ESRD, a doubling of the creatinine level or death from renal caused by 34% [191]. This result indicates that SGLT2 inhibitors might be the new hope of the patients with DN after the use of renin-angiotensin system blockers over the past 18 years. The underlying mechanism may be related to its antisenescence of renal cells resulting from the fact that SGLT2 increased the expression of senescent markers in proximal tubules [76] and endothelial cells [192] in DM, indicating that SGLT2 inhibitors might retard renal accelerated aging in DM to preserve kidney function. Other antidiabetic agents reported to protect against accelerated aging are dipeptidyl peptidase 4 (DPP4) inhibition and GLP-1 receptor agonists, which act on the modulation of incretin that protect against age-related diseases including DN [193]. Actually, DPP4 inhibition demonstrated clear antiaging effects.

The lifespan of klotho^−/−^ mice was prolonged, and their body weight was significantly related to greater kidney weight in the intervention of linagliptin [194]. Although no conclusive evidence has demonstrated that DPP4 inhibition improves diabetic kidney lesions, it may control blood glucose and albuminuria as well as be tolerated in patients with DM and CKD, indicating potential renal benefits [195]. It has been reported that rectification of the imbalance between DPP4 and GLP-1 is helpful to vascular aging [196]. Thus, besides DPP4 inhibition, GLP-1 receptor agonists have also shown antiaging potential. GLP-1 receptor agonists showed renoprotection independent of glycemic control, such as inhibition of cellular apoptosis, inflammation, and oxidative injury via the upregulation of Sirt1 [197]. Nevertheless, the renoprotective effect had seemingly acted indirectly on the kidney but was associated with a systemic immunomodulatory effect [198].

PPAR*γ* expresses a low level in kidneys, and it has been shown that its expression and activity reduce during aging and results in the loss of aging-associated function [199]. Pioglitazone presented antiaging via the upregulation of Sirt1 and Klotho, decreased the p53 protein level in aged ApoE^−/−^ mice [200], and alleviated aging-related renal injury via modulation on mitochondrial function [201]. Clinical studies suggested that low-dose pioglitazone was an effective renoprotective method in DN [202, 203]. As a result, pioglitazone showed its potential protection against accelerated senescence in DN.

Considering that oxidative stress plays a major role in the progress of DN and accelerates the kidney aging, chronic antioxidant supplements are eagerly sought after for their long-term benefits. Antioxidants (including vitamin C, vitamin E, and zinc) may protect against early renal damage [204]. High-dose vitamin E supplements in the treatment of DN resulted in a notable decrease in urine protein, which may be partly associated with the alleviation of autophagic stress in TECs [117, 205]. Nicotinamide adenine dinucleotide (NAD) functions as a coenzyme in redox reactions and mediates many biological processes, including metabolism and aging, as well as metabolic diseases like DM [206, 207]. As a hallmark of aging, NADs are related to the inducing of autophagy, repairing DNA and activation of Sirt1 and NADs, which are regulated by AMPK [208]. The age-dependent decrease of NADs happens in many tissues, including kidneys [206]. NAD precursors can delay aging and counteract a broad spectrum of age-related diseases. It has been reported that NAD replenishment contributed to retarding the renal lesion in diabetic rats. However, the precision of NAD+ supplementation is needed due to the regulation of SASP leading to tumor-promoting effects [209]. Actually, a number of studies are focused on antioxidant supplementation in the treatment of DN, but conclusive evidence is still lacking to demonstrate their long-term clinical benefits.

Owing to the fact that fewer senescence cells can even lead to reduced survival in older individuals, senotherapies, such as selective elimination of senescent cells (senolytics) or the disruption of the senescent cells' secretome (senostatics), are gaining significant attention from researchers to retard the progression of aging-related diseases [210, 211]. So far, dozens of senolytics have been reported, which have been considered adjunctive therapies for aging-related diseases such as tumors [212], idiopathic pulmonary fibrosis [213], Alzheimer's disease [214], and renal disease [215]. These reports suggest that senolytics may be a beneficial supplementary therapy for DM patients with chronic kidney injuries. A recent clinical study reported that 3 d of oral Dasatinib and Quercetin alleviated adipose tissue senescent cell burden and decreased skin epidermal p16^INK4A+^ and p21^CIP1+^ cells and circulating SASP factors in patients with DN, indicating that senolytics relieved senescent cell burden [216]. Hence, it is hypothesized that a similar intervention could be complementary to clinic therapy in the treatment of DN in the future. Actually, the application of senotherapies in aging-related disease remains uncertain [217], including their specific-kidney protection, so more clinical and further studies are needed.

Simultaneously, traditional Chinese medicine (TCM) is another popular option for DN because of its definite curative effect. The renal protection offered by Shenkang injection, a classic compound prescription, has also been demonstrated to retard high glucose-induced senescence of renal tubular cells [218]. Some extracts of herbs have exhibited antiaging properties, such as curcumin [219, 220], a glycoprotein isolated from Fupenzi [221], tea polyphenols [222], the flavonoid 4,4′-dimethoxychalcone (DMC) [102], and berberine [223]. These results imply that TCMs appear to have potential advantages to protect renal function against kidney aging in DM in the future. The potential mechanisms of drugs with antiaging properties applied to DN are summarized in Table 2.

## 8. Conclusion

Accelerated senescence of kidneys plays a vital role in DN progression. To delay renal lesions in DM or DN patients, accelerated or premature kidney aging must be delayed. The underlying mechanisms of accelerated kidney aging in DM or DN remain complex and multiple. Hyperglycemia, inflammation, oxidative stress, and hypertension induce renal inherited cellular senescence and the downregulation of antiaging proteins, such as Sirt1 and Klotho, and the inactivation of the lysosome-dependent autophagy pathway.

Antihyperglycemia is the most important factor in the treatment of DM and the prevention of DN. Metformin, pioglitazone, and the inhibitor of SGLT2 have been reported to exhibit antisenescence effects independent of antihyperglycemia. Owing to its certain renoprotection for DN patients, inhibitors of SGLT2 are given priority to consideration besides ACEI/ARBs. Additionally, metformin and pioglitazone should be more considered in the treatment of DM and in the early stage of DN. In addition, for DM or DN patients, antioxidant-rich food and antisenescence-compound-rich food are recommended. However, it should be noted their intake should be considered according to their sugar content. Concurrently, moderate calorie restriction and a level of exercise are encouraged for patients with DM and early DN.

Autophagy is the common downstream of aging-related pathways and is central to DN progression. Unfortunately, the regulation and activity mechanisms of autophagy remain incompletely understood with respect to DN. It has been proved that Sirt1 has been regulating autophagy through the deacetylation of Atg5, Atg7, and Atg8 [224]. Autophagy extended the lifespan of mice knock-out Klotho [225], and Klotho attenuated renal lesions by regulating the autophagy clearance [226]. Based on these studies, we identified a key link between antiaging proteins like Sirt1, Klotho in DN, but further studies are still necessary to illustrate how the antiaging proteins regulate the autophagy and the exact sites for autophagy modulation.

## Figures and Tables

**Table 1:** Comparison between SASP and DN-associated secretory phenotype.

SASP factors	Secretory profile for senescent cells [67, 227]	DN-associated secretory phenotype	
Cytokines			
IL-1*α*,-1*β*,-2,-4,-6,-10,-13-17,-18,-20	↑	↑	[228–239]
TNF-*α*	↑	↑	[229, 232, 233, 237–239]
TWEAK	↑	↑	[240]
ICAM-1	↑	↑	[230, 233, 241–244]
VCAM-1	↑	↑	[232]
Chemokines			
CXCL-1,-5,-7	↑	↑	[233, 243]
CCL-2,20	↑	↑	[233]
CCL-4,-5	↑	↑	[230, 243, 245]
IL-8	↑	↑	[236]
MCP-1	↑	↑	[235, 236, 238, 243–246]
MIP-1,-2	↑	↑	[243, 244]
Eotaxin	×	↑	[241]
Other inflammatory factors			
GM-CSF	↑	↑	[233]
G-CSF	×	↑	[234, 237]
IFN-*γ*	×	↑	[229]
Growth factors and regulators			
IGFBP-3, -7	↑	↑	[247, 248]
TGF-*β*	↑	↑	[96, 117, 234, 242, 246]
VEGF	↑	↑	[230, 233]
PDGF	↑	↑	[233]
FGF-2, 23	↑	↑	[249, 250]
Proteases and regulators			
MMP-2, -9,	↑	↑	[232, 251, 252]
TIMP-1	↓or ×	↓	[251]
TIMP-2	↑	↑	[242]
PAI-1	↑	↑	[230, 232, 253]
Cathepsin B	↑	↑	[106, 117]
Insoluble factors (ECM)			
Fibronectin	↑	↑	[239, 246, 253]
Collagens	Altered	↑	[231, 234, 239, 242, 246, 253]
Other factors			
iNOS	↑	↑	[235]
ROS	Altered	↑	[96, 239]
COX-2	↑	↑	[235]
NOX-4	↑	↑	[254]
SOD	↓	↓	[96, 235, 238, 239, 254]
MDA	↑	↑	[96, 235, 238, 239, 254]

Abbreviations: SASP: senescence-associated secretory phenotype; IL: interleukin; TNF-*α*: tumor necrosis factor *α*; TWEAK: apoptosis of tumor necrosis factor-like weak inducer; ICAM-1: intercellular adhesion molecule 1; VCAM-1: vascular cell adhesion molecule 1; CXCL: C-X-C-motif chemokine ligand; CCL: C-C-motif chemokine ligand; MCP: monocyte chemoattractant protein; MIP: macrophage inflammatory protein; GM-CSF: granulocyte-macrophage colony-stimulating factor; G-CSF: granulocyte colony-stimulating factor; IFN-*γ*: interferon-*γ*; IGFBP: insulin-like growth factor binding protein; TGF-*β*: transforming growth factor -*β*; VEGF: vascular endothelial growth factor; PDGF: platelet-derived growth factor; FGF: fibroblast growth factor; MMP: matrix metalloproteinase; TIMP: tissue inhibitors of metalloproteinases-1; PAI-1: plasminogen activator inhibitor -1; iNOS: inducible nitric oxide synthase; ROS: reactive oxygen species; COX-2: cyclooxygenase-2; NOX-4: NADPH oxidase-4; SOD: superoxide dismutase; MDA: administration; ^∗^Upward arrows, crosses, and downward arrows show secretory increase, no change and decrease in senescence, respectively.

**Table 2:** Examples of potential mechanisms underlying renal benefits of drugs with antiaging effects.

Drugs	Beneficial effects	Mechanisms	In vivo	In vitro	Ref.
Resveratrol	Attenuation of renal fibrosis	Regulation of AMPK/NOX4/ROS signaling	db/db mice		[255]
Resveratrol	Renoprotection	Enhancement of hypoxia-induced autophagy via Sirt1	STZ-induced diabetic rats,	Hypoxic-condition-induced rat proximal tubular epithelial cells NRK-52e	[110]
Resveratrol	Suppression of renal inflammation and mesangial cell proliferation	Modulation on Akt/NF-𝜅B pathway	STZ-induced diabetic rats;	HG-induced rat mesangial cells	[256]
Resveratrol	Regulating oxidative stress and mitochondrial function	Modulation of the Sirt1/FoxO1 signal pathway	STZ-induced diabetic rats	HG-induced rat mesangial cells	[165, 257]
Resveratrol	Renoprotection	Extenuating the oxidative stress and downregulation of RAGE expression	STZ-induced diabetic rats		[258]
Resveratrol	Ameliorating lipotoxicity, oxidative stress, apoptosis, endothelial dysfunction; glomerular matrix expansion and inflammation	Activating the AMPK-Sirt1-PGC-1*α* axis and PPAR*α* through increases in AdipoR1 and AdipoR2 expression	db/db mice	HG-induced human glomerular endothelial cells and NMS2 mesangial cells	[259, 260]
Resveratrol	Protection on podocytes	Activation of autophagy involved with miR-383-5p	db/db mice	HG-induced human podocytes	[261]
Resveratrol	Protection on mesangial cells	Negative regulation of the p38 MAPK/TGF-*β*1 pathway	STZ-induced diabetic rats	HG-induced rat mesangial cells	[167]
Resveratrol	Protection on podocytes	Against apoptosis by increasing autophagy via miRNA-18a-5p expression	db/db mice	HG-induced human podocytes	[262]
Resveratrol	Effect on endoplasmic reticulum stress	Reducing expressions of 78 kDa glucose-regulated protein (GRP78), protein kinase RNA-like endoplasmic reticulum kinase (PERK), and activating transcription factor 4 (ATF4) and C/EBP-homologous protein (CHOP)	STZ induced diabetic rats		[263]
Resveratrol	Protection on podocytes	Reducing oxidative damage and apoptosis of podocytes via Sirt1/PGC-1*α* mitochondrial protection		HG-induced immortalized mouse podocytes	[164]
Resveratrol	Renoprotection and reducing albuminuria	Suppression of the angiotensin II (Ang II)/angiotensin II type 1 receptor (AT1R) axis and enhancing the angiotensin 1-7 (Ang 1-7)/Mas receptor (MasR) axis; anti-inflammation and oxidative stress	Aged C57BL/6 mice		[30]
Resveratrol	Regulation on endothelial dysfunction	Modulation of Sirt1 and PPAR*γ*	db/db mice		[166]
Metformin	Restore the insulin responsiveness of podocytes	Regulating Sirt1 and AMPK activities		HG-induced rat podocytes	[182]
Metformin	Exhibiting an anti-apoptotic impact on podocytes	Activation of AMPK and inhibition of mTOR signaling		HG-induced immortalized human podocytes	[178]
Metformin	Renoprotective effect	Increasing SOD activity and decreasing malondialdehyde level; decreasing the expression levels of TGF-*β*1	STZ-induced diabetic rats		[264]
Metformin	Improving diabetic tubulopathy	Increasing in PGC1*α* activity by modulating mitochondrial dynamics and autophagy	STZ-induced diabetic mice,	HG-induced human renal proximal tubular epithelial cell line HKC8	[188]
Metformin	Against proteinuria cytotoxicity	Suppression of Akt and mTOR activation, inhibition of EMT and apoptosis and augmentation of autophagy and ER defense response through AMPK-independent and AMPK-dependent mechanisms		Albumin-induced rat renal proximal tubular cells	[187]
Metformin	Protection on podocytes	Upregulating the renal tissue nephron expression	STZ-induced rats		[180]
Metformin	Protection on podocytes	Increases extracellular ATP concentration, leading to activation of P2 receptors and consequent modulation of the podocytes' metabolism through AMPK and NAD(P)H oxidase		HG-induced mouse podocytes	[179]
Metformin	Alleviation of cell senescence	Downregulation of Connexin43 via activation of AMPK and the inhibition of mTOR		HG-induced primary rat glomerular mesangial cells	[189]
Metformin	Alleviation of high-glucose-induced oxidative stress	Regulating p-p38MAPK protein expression		HG-induced rat glomerular mesangial cells	[265]
Metformin	Alleviation of inflammation	Inhibits nuclear factor-*κ*B activation and inflammatory cytokines expression including monocyte chemoattractant protein-1 (MCP-1), intercellular adhesion molecular depend on AMPK		HG-induced rat glomerular mesangial cells	[183]
Metformin	Inhibition of apoptosis and inflammatory and fibrotic reactions in tubular cells	Reducing ROS generation via suppression of RAGE expression through AMP-activated protein kinase activation		AGEs induced human proximal tubular epithelial cells	[266]
Metformin	Relieving oxidative stress, slowed down abnormal cell proliferation	Enhancing autophagy and through Sirt1/FoxO1 pathway via AMPK	STZ-induced diabetic rats;	HG-induced rat mesangial cells,	[185, 186]
Metformin	Exerting anti-inflammatory	Upregulating GLP-1R expression via AMPK	db/db mice	HG-induced rat mesangial cell line (HBZY-1)	[184]
Metformin	Attenuating hypoxia	Reducing uncoupling protein-2- (UCP2-) mediated mitochondrial proton LEAK	STZ-induced diabetic rats		[267]
Metformin	Alleviating cell senescence	Reducing p21 expression by activating AMPK.		Human embryonic kidney (HEK293) cell line	[190]
Metformin	Improving epithelial-to-mesenchymal transition	Inhibiting early growth response- (Egr-) 1; inhibiting MCP-1 expression via BMP and activin membrane-bound inhibitor- (BAMBI-) mediated inhibition of ERK1/2		TGF-*β*1-induced rat renal tubular epithelial cell line (NRK-52E)	[268, 269]
GLP-1	Protection of podocytes	Against apoptosis, inhibition reactive oxygen species production and proinflammatory cytokine secretion, through Sirt1 activation		HG-induced mouse podocytes	[197]
GLP-1R agonist (Exendin-4)	Against renal fibrosis	Inhibiting the transfer of extracellular vesicle miR-192		HG-induced renal tubular epithelial cells	[270]
GLP-1R agonist (Exendin-4)	Inhibiting cell proliferation and fibronectin secretion	Reversing ERK phosphorylation and enhancing expression of mTOR via AMPK		HG-induced rat mesangial cells	[271]
GLP-1 analog (liraglutide)	Against renal inflammatory and protection on endothelial cells	Inhibiting STAT3/JAK2 expression via SIRT1	db/db mice	AGEs- or HG-induced endothelial cells	[272]
GLP-1R analog (liraglutide)	Ameliorating early renal injury	Increasing the expression of FoxO1 mRNA and reducing renal phosphorylation levels of Akt and FoxO1 protein	STZ-induced diabetic rats		[273]
GLP-1R analog (liraglutide)	Renoprotective effect	Inhibiting autophagy and apoptosis dependent on GLP-1R		HG-induced human renal tubular epithelial cell line (HK-2)	[274]
GLP-1 analog (liraglutide)	Against oxidative stress and albuminuria	Via a PKA-mediated inhibition of renal NAD(P)H oxidase	STZ-induced diabetic rats	HG-induced human mesangial cells	[275]
DPP 4 inhibitor (sitagliptin)	Attenuation of glomerular lesions	Alleviation of oxidative injury	STZ-induced diabetic rats		[276]
SGLT2i (dapagliflozin)	Attenuation of renal fibrosis	Elevating O-GlcNAcylation and tubular hypoxia	STZ-induced diabetic rats	HG-induced human proximal tubular epithelial cell line (HK-2)	[277]
SGLT2i (dapagliflozin)	Against inflammation and postponing the progression of renal injury	Inhibition of HMGB1-RAGE-NF-*κ*B signaling pathway		HG-induced human proximal tubular epithelial cell line (HK-2)	[278]
SGLT2i (canagliflozin)	Against renal inflammation, extracellular matrix turnover and fibrosis	Reduction in TNFR1, IL-6, MMP7 and FN1		HG-induced human proximal tubular epithelial cell line (HK-2)	[279]
SGLT2i (Ipragliflozin)	Improvements in glomerular damage	Normalizing the levels of accumulated tricarboxylic acid cycle intermediates and increased oxidative stress	db/db mice		[280]
SGLT2i (Empagliflozin)	Anti-inflammatory and antifibrotic effects	Suppressing AGE-RAGE axis	STZ-induced diabetic rats		[281]
Pioglitazone	Reprotection in DM	Decreasing expression of hypoxia-inducible factor-1a (HIF-1a) and vascular endothelial growth factor (VEGF)	STZ-induced diabetic rats		[282]
Pioglitazone	Ameliorating aging-related renal injury	Increasing klotho, decreasing oxidative stress, and mitochondrial injury; regulating p66^Shc^ phosphorylation, which integrates many signaling pathways that affect mitochondrial function and longevity, by reducing protein kinase C	Aging male Sprague-Dawley rats		[201]
Dasatinib and quercetin	Decreasing human senescent cell burden	Alleviating adipose tissue senescent cell burden, decreasing skin epidermal p16^INK4A+^ and p21^CIP1+^ cells and circulating SASP factors in patients with DN	Human tissues		[216]

Abbreviations: STZ: streptozotocin; DN: diabetic nephropathy; HG: high glucose; GLP-1: glucagon-like peptide-1; DPP4: dipeptidyl peptidase 4; SGLT2i: sodium-glucose cotransporter-2 inhibitor; AMPK/NOX4/ROS: AMP-activated protein kinase/NADPH oxidase-4/reactive oxygen species; Akt/NF-*κ*B: protein kinase B/nuclear factor kappa-B; FoxO1: forkhead box O 1; PGC-1: peroxisome proliferator-activated receptor- (PPAR-) *α* coactivador-1; AdipoR: adiponectin receptor protein; MAPK: mitogen-activated protein kinase; TGF-*β*: transforming growth factor-*β*: mTOR: mammalian target of rapamycin; SOD: superoxide dismutase; EMT: epithelial-mesenchymal transdifferentiation; RAGE: receptor for advanced glycation end products (AGEs); HMGB1: high mobility group box 1-receptor; ERK: extracellular signal-regulated kinase; STAT3: signal transducer and activator of transcription; JAK2: janus kinase 2; TNFR1: TNF receptor 1; MMP7: matrix metalloproteinase 7; FN1: fibronectin 1.
